# 7-Tesla PET/MRI: A promising tool for multimodal brain imaging?

**DOI:** 10.3934/Neuroscience.2022029

**Published:** 2022-12-16

**Authors:** Arosh S. Perera Molligoda Arachchige

**Affiliations:** Department of Biomedical Sciences, Humanitas University, Milan, Italy

## Dear Editor,

The dawn of ultra-high field imaging has undoubtedly shown its value in both research and clinical practice especially in cognitive neuroimaging. Scientists have long made an effort to incorporate the anatomical, functional, blood flow information provided by MRI with metabolic, molecular information provided by PET in order to establish new connections among them and have so far made a huge progress. This is because in PET/CT, the CT examination adds a substantial X-ray dose which has been shown through several studies to easily rise above the dose from an applied PET isotope to even result in altered immune responses or biological pathways in animal models, and is significantly large enough to cause a radiotherapy treatment like effect in studies investigating the effectiveness of novel anti-tumor drugs potentially obscuring their results [1]. Furthermore, PET/MRI offers better soft tissue contrast over PET/CT in specific regions such as the brain parenchyma and has shown to be even greater with higher field strengths since gains in signal-to-noise or contrast-to-noise ratio and greater spatial resolution have allowed finer structures to be visualized and smaller physiological effects to be detected compared to lower field systems. Also, PET/MRI provides greater flexibility in terms of the plurality of sequences [2].

Mayo Clinic was the first centre in North America to use clinical 7-tesla MRI, after the system was approved in 2017. Currently, Mayo Clinic uses both positron emission tomography (PET) scans and 7-tesla MRI to image individuals with epilepsy to identify potential candidates for surgical treatment. Here, they use 7T imaging if initial PET scans and 3-tesla MRI indicate metabolic abnormalities associated with seizure-origin sites as well as potential suspect lesions which according to them has allowed to detect 20–30% of lesions that went undetected with 3T [3]. This is an example of asynchronous multimodality imaging which aims to combine morpho-functional information by acquiring images at different times, and fuse them through digital image manipulation techniques. This however comes with a set of constraints, mainly conditioned by the different positioning of the patient in the two scans acquired at different times in separated machines [4]. Even if the two scanners are mounted in-line in a common gantry and with a single patient couch, attenuation correction will be required and thus results in lengthy transmission scans and increased patient throughputs [5]. On the contrary, synchronous image acquisition being a one-stop-shop procedure allows to achieve consistency in time and space. It has benefits in terms of patient comfort, decreased demand on instrumental and administrative resources and could be especially advantageous in dementia patients with reduced ability to cooperate [4],[5]. Although there are clear differences between the two, the motivation to integrate PET and MRI was the huge success derived from PET/CT. Such PET/MRI technology has been efficiently utilized at conventional field strengths in the context of several neurological conditions such as Alzheimer's disease, Parkinsonian syndromes, multiple sclerosis, brain tumors, epilepsy, etc [6].

So, in what ways does the incorporation of ultra-high field imaging benefit? A study by Hammer et al. showed that magnetic field strengths above 5T can further improve PET image quality by reducing the mean free positron path between emission and annihilation and this has been made possible since latest PET/MRI developments use solid-state light detectors that can be operated even at high magnetic fields [7]. Indeed, the 7T PET-MRI, the very latest innovation in preclinical imaging of animal models is a PET system coupled with (a cryogen-free) 7T MRI that enables simultaneous PET and MR imaging data acquisition. To our knowledge, 7T PET/MRI is currently manufactured exclusively by “MR Solutions (UK)” and is already available in a few countries notably the US, Norway, and Israel [8]. Additionally, in a study by Cho et al. where a PET/MRI system consisting of a HRRT (high-resolution research tomograph; a human brain PET scanner) and a 7T MR scanner were operated for in-vivo visualization of thalamic subnucleus quantitative glucose metabolism, the high-resolution, high-contrast images of the 7T MR component scanner enabled in-detail discrimination of the thalamic nuclei, allowing precise localization of the corresponding in vivo metabolic activities obtained by the HRRT. This PET molecular imaging system could be useful in investigating mechanisms of pain or it could be extended to D2 receptor distribution in each thalamic nucleus in imaging of Parkinsonian syndromes, and many more [9]. These are just a few studies to mention, and many more studies are yet to come with the adequate distribution of the 7T PET/MRI facilities globally. If this tool is to have widespread impact outside a small group of academic sites, training the next generation of technicians and interpreters is a challenge which will have to be met. According to Catana et al. one of the main challenges, the limited space available inside the bore of standard MR systems to integrate the PET detectors, has been solved by introducing larger bore diameters that provided adequate space [10]. However, we found no evidence explaining why 7T PET/MRI with large bore sizes in order for them to be used in the clinic are not yet available. We assume that it has been due to the lack of adequate studies highlighting the clinical potential of this new multimodality imaging tool. In addition, more research is needed to determine the cost effectiveness of PET-MR technology. We are confident that it will be possible to translate these technologies used in preclinical studies to human PET/MRI scanners with 7-T or higher-field magnets through further technologic developments and appropriate modifications, which will lead the next generation of molecular imaging.
